# Supporting Youth and Families in Gaza: A Randomized Controlled Trial of a Family-Based Intervention Program

**DOI:** 10.3390/ijerph19148337

**Published:** 2022-07-08

**Authors:** Laura E. Miller-Graff, E. Mark Cummings

**Affiliations:** 1 Psychology and Peace Studies, University of Notre Dame, 107 Haggar Hall, Notre Dame, IN 46556, USA; 2 Department of Psychology, University of Notre Dame, 118 Haggar Hall, Notre Dame, IN 46556, USA; cummings.10@nd.edu

**Keywords:** political violence, Palestine, mental health and psychosocial support, emotional security, cognitive behavioral, emotion regulation, adjustment, adolescents

## Abstract

**Background:** A total of 450 million children are now living in active conflict zones. The negative consequences for children are significant and long lasting. In response to the urgent need for sustainable interventions for children and families, the current study evaluated a brief (12 hr, 8 session) family-based coping and support program, *Promoting Positive Family Futures* (PPFF), in Gaza. **Methods:** Families (*n* = 68, mother/father/adolescent triads) were randomized into the PPFF intervention or another lengthier (50 hr, 25 session) locally well-established psychosocial support program (treatment as usual; TAU). **Results:** Improvements were found for both conditions for paternal and maternal depression, emotion regulation using cognitive reappraisal, family-wide emotional security, and adolescent adjustment. Effect sizes were medium to large (*d* = 0.35–1.27). Fathers in the PPFF condition reported lower depression and higher emotion regulation using cognitive reappraisal at post-test than did fathers in the TAU condition. Mothers in the PPFF condition reported higher levels of emotion regulation using cognitive reappraisal at post-test than did mothers in the TAU condition. PPFF was also indirectly associated with improved depression at post-test for both mothers and fathers via improvements in emotion regulation using cognitive reappraisal and with adolescent adjustment at six months via improvements in maternal emotion regulation using cognitive reappraisal. **Conclusions:** These findings suggest that the PPFF intervention has many benefits comparable with a longer and locally well-established program. PPFF was also associated with unique positive implications for family-wide adjustment over time. Support was also identified for transdiagnostic processes of improvement associated with the PPPF intervention consistent with the theoretical models informing the approach.

## 1. Introduction

At the end of 2020, more than 33 million children were forcibly displaced, and an estimated 452 million children were living in active conflict zones [1,2]). The consequences of such experiences for children are significant and long lasting, making violence one of the gravest preventable threats to the health and wellbeing of children worldwide [3]. In contexts characterized by chronic conflict, research has identified numerous factors contributing to the risk for psychological distress in children, including the experience of multiple and overlapping types of violence (e.g., direct, structural, and cultural violence) [4]), a fractured sense of emotional security in community relationships [5], and social narratives that forward an “ethos” of conflict [6]. Despite the known effects of political violence and armed conflict on children [7], few mental health and psychosocial support programs (MHPSS) have been able to demonstrate compelling evidence of effectiveness for children and youth living in these settings [8], and many existing programs fail to substantively engage family members in care [9]. Another gap in intervention research in contexts of political violence and armed conflict is the inclusion of fathers in family-based interventions, despite evidence that fathers and fathering may be particularly vulnerable [10] and that the presence of fathers is beneficial for adolescents [11]. Moreover, few intervention studies in conflict-affected settings have adequately identified or tested the mechanisms hypothesized to underlie treatment effects. Such process-oriented research is increasingly understood as critical to advancing intervention research. To address these gaps in intervention research in contexts of political violence and armed conflict, the current study examines the effects of a brief program, the *Promoting Positive Family Futures* (PPFF), designed to support family-wide communication and coping in the context of ongoing conflict in Gaza using a randomized controlled trial design.

In previous research on a large representative sample of youth in the West Bank and Gaza, Dubow and colleagues (2009) [12] found that 91% of children had witnessed violence at school, 89% had witnessed violence in the community, and 61% experienced the loss of or injury to a friend or family member. In the Gaza Strip, specifically, severe forms of violence and trauma are common: a recent study found that 89.4% of youth reported exposure to 6 or more traumatic events, and 48.5% reported exposure to 10 or more events, the most common of which were seeing mutilated bodies on TV, hearing shelling and being forced to leave home due to shelling [13]. Recent escalations of violence in Gaza have included airstrikes and land incursions as well as mass violence against protesters. In addition to these direct forms of violence, Gazans experience numerous forms of structural violence and deprivation as a result of the occupation. For example, travel for residents is severely restricted due to a 15-year air, land, and sea blockade that controls the movement of people and goods both in and out of Gaza. Thus, children and families in the Gaza Strip have ubiquitous exposure to sociopolitical violence [14].

Supporting youth and families in the context of such ubiquitous violence is complex. Youth exposure to violence is associated with a broad range of short- and long-term adverse effects on mental health, including depression, anxiety, behavior problems, and traumatic stress [12,15,16]. Previous work on mental health and psychosocial support (MHPSS) in the West Bank and Gaza has been criticized for failing to account for several key features of the conflict and its effects on children and families. First, it has been noted that distress in the wake of violence is normative, and second, that care often does not take fully into account the communal nature of trauma and the need for justice [17]. Such critiques are also borne out in systematic reviews of the treatment literature in the West Bank and Gaza, which demonstrate that most programs that have been rigorously evaluated in the region are individualized treatments for posttraumatic stress [18]. Thus, the lack of MHPSS that robustly addresses psychological distress, broadly construed, and that includes broader social systems and processes is a gap relative to the specific evidence-basis in the West Bank and Gaza but also in the global literature more broadly.Few empirically evaluated programs for youth in conflict-affected settings engage robustly with family systems and community strengthening [9]. This gap in the MHPSS literature is concerning given the widely demonstrated importance of family and community functioning for youth mental health in contexts of political violence [7] and research demonstrating the importance of parental engagement in treatment [19]. Research in the West Bank and Gaza has also demonstrated that ongoing violence is associated with more challenges in youths’ family functioning [15]. Research from other contexts suggests that politically-motivated violence—to an even greater extent than apolitical forms of community violence—is associated with higher rates of family conflict and greater reports of youth-reported emotional insecurity in the family [20,21]. Together this research suggests that parental engagement is likely not only to enhance MHPSS for adolescents, but integrated supports for children and families is consistent with systemic approaches to care that recognize the broad and diverse effects of violence.

The theoretical conceptualization of MHPSS in conflict-affected settings must therefore recognize both the individual and systemic effects of violence, acknowledge that distress in light of violence is not pathological, and demonstrate an awareness of how MHPSS intersects with other community and social processes aimed at ending violence. Resilience theory, which focuses on how individuals actively navigate complex social systems to facilitate and promote adaptation in the face of significant adversity [22], provides a useful overarching lens for approaching MHPSS in conflict-affected settings. First, it recognizes that psychological distress in the wake of chronic violence is normative [23] and that systems-focused approaches that frame risk and resilience as dynamic and interactive more fully articulate how families can be supported in the context of chronic threats [22,24]. Moreover, local understandings of resilience (*sumud*) give insight into the ways in which Palestinians living under occupation conceptualize the role of resistance and dignity in daily life [25]—critical concepts for preventing MHPSS from becoming a form of palliative care.

Within this broader theoretical framework, emotional security theory and empirical work on the maintaining factors of individual psychological distress can be integrated to inform process-oriented approaches to supporting both familial and individual health. Emotional security refers to children’s sense of protection, safety, and security in their family and community [26,27]. Youths’ emotional security in the family system, which is reflected by processes of emotion and behavior regulation and cognitive appraisal, has been consistently linked with numerous aspects of youth adjustment, including peer problems, anxiety, and depression [28]. Importantly, emotional security in the family system is influenced by the psychological health of all family members. For example, prospective research has documented the negative effects of parental depression on both children’s emotional security and psychological health [29,30]. Brief, family-based approaches to intervention to support adolescent and family emotional security and communication have demonstrated effectiveness in previous research. Specifically, such programs have shown that intervention-related improvements in constructive conflict are associated with lower adolescent internalizing at six-month follow-up [31].

Together this research suggests that in contexts with ongoing sociopolitical violence, addressing factors contributing to youth and parental psychological distress, in addition to supporting positive family-wide processes is likely to be beneficial. Psychological research has identified emotion dysregulation and cognitive appraisal as two key processes underlying the emergence and maintenance of psychological distress in the context of adversity, including in the West Bank and Gaza [32,33,34]. Both of these processes are directly addressed in cognitive behavioral therapy, which has demonstrated effectiveness for a broad range of presenting problems in numerous contexts and with diverse populations, including in conflict-affected settings, in the Middle East, and with both adolescents and adults [35,36]. Moreover, there is substantial evidence that even brief approaches to care are able to produce meaningful change. For example, Barron and colleagues (2013) [35] identified positive changes in posttraumatic stress symptoms, grief, and depression associated with a brief, 5-session CBT intervention for adolescents. Accordingly, brief, targeted modules based on core elements of CBT are delivered as one dimension of PPFF (see sessions 4 and 5, below).

Drawing together both basic and translational research from these three theoretical models (emotional security theory, resilience theory, and cognitive behavioral therapy), there is substantial evidence to suggest the promise and relevance of their integration to target key underlying transdiagnostic mechanisms of change across the family system. Intervention approaches are increasingly concerned with identifying transdiagnostic mechanisms of risk for adjustment problems in children and adults [32,37]. It has been noted that few studies have identified or tested mechanisms of treatment from a process-oriented perspective [7]. Identifying and testing such mechanisms are an important area of growth for the MHPSS literature [38]. Thus, the current study aims to address a pressing gap in the research literature through a preliminary evaluation of the effectiveness and potential mechanisms of change of a newly developed, family-based intervention program.

## 2. The Current Study

The current study draws upon rich theoretical work, including emotional security theory, resilience theory, and cognitive behavioral theories, as well as context-specific empirical work to address a key gap in the research literature on political violence, armed conflict, and youth adjustment [7]. Namely, the current study conducts an evaluation of a brief, family-based intervention—the *Promoting Positive Family Futures* (PPFF) program—designed to synergistically support the individual mental health of family members as well as family-wide emotional security and communication. These co-occurring processes of change are framed within a dynamic systems framework that understands development and resilience as interactive and multiply determined. The current study used a randomized controlled trial design, with three time points of assessment (pre-test [T1], post-test [T2] and six-month follow-up [T3]). The intervention (PPFF), which is 8 sessions in duration and includes mothers, fathers, and adolescents, is compared against a treatment as usual (TAU) condition consisting of a 25-session, adolescent-only intervention that is well-established and commonly offered in the location of the study. Given that the PPFF intervention, while rooted in previous both basic and translational work, is newly developed, a pilot trial was selected as an appropriate first step to evaluate its effectiveness. Pilot trials are relatively small-*n* studies designed to assess both feasibility and preliminary evidence of effectiveness in order to determine whether a full-scale trial is merited [39]. As such, the current study did not aim to achieve full statistical power to detect small differences between groups, but rather had sufficient power to determine the feasibility of the research design, program, and to gather initial evidence of effectiveness and possible mechanisms of change. Hypotheses were as follows:

(1) Both treatment groups (PPFF and TAU) would demonstrate positive within-group change from baseline to post-test (T1–T2) and from baseline to follow-up (T1–T3), including in parental depression, parental emotion regulation using cognitive reappraisal, family-wide emotional security, adolescent adjustment, and adolescent resilience.
(2) Despite the advantage in amount of treatment exposure for the TAU, the PPFF intervention would demonstrate better effectiveness as compared with the TAU. That is, when differences between program effects exist, it was hypothesized that they would be in favor of the PPFF program.
(3) Consistent with the integrated theoretical framework of the current study, improvements in parental depression would be indirectly explained by improvements in emotional regulation using cognitive reappraisal.
(4) Consistent with common processes identified across the integrated theoretical models under study, improvements in adolescent adjustment would be indirectly explained by improvements in parental emotion regulation using cognitive reappraisal and parental depression.

## 3. Materials and Methods

### Participants

Participants included *n* = 68 families in Gaza City, Gaza. In order to be eligible to participate, families had to (1) have at least one adolescent child between the ages of 13 and 16, (2) be willing for both parents to participate in the intervention, if parents were currently married, and (3) live in a community catchment area for Catholic Relief Services (CRS). There was a relatively even balance of participating male (57.35%) and female (42.65%) adolescents, who ranged in age from 13 to 16 (*M* = 12.03, *SD* = 1.06). Mothers ranged in age from 30 to 59 (*M* = 47.03, *SD* = 6.84), and fathers ranged in age from 35 to 65 (*M* = 41.14, *SD* = 6.61). Approximately half of the families in the sample had fathers who were currently working (45.59%). A small number of mothers (4.41%) and adolescents (5.58%) also reported that they were currently working. Average family income for the past month was 849.04 NIS (*SD* = 565.90), which is the equivalent of approximately $262 USD. There was significant variation in previous educational attainment. Of participating fathers, 39.71% had completed primary education or less, 32.35% had completed some secondary education, 13.24% had completed their secondary education, 5.88% had completed some college, 2.94% had completed both college and some graduate school, and 5.88% held a graduate degree. Of participating mothers, 48.53% had completed primary education or less, 23.53% had completed some secondary education, 13.24% had completed their secondary education, 5.88% had completed some college, 4.41% had completed both college and some graduate school, and 4.41% held a graduate degree.

## 4. Procedures

### 4.1. Site Selection and Recruitment

Prior to recruitment, CRS local and international staff engaged with community members and leaders in a series of community meetings in order to determine the best locations for services and to discuss the best ways to explain services and the proposed research design to families. Following these meetings, a community center in Middle Gaza was identified, and community spaces easily accessible to families were identified and rented. Following this, community meetings were held to explain the project, the program, the eligibility requirements, and registration information. Families were given the opportunity to ask questions and a number to call with any questions or concerns that they would like to follow-up on after the session.

### 4.2. Study Preparation

Following site selection, the research team, the CRS leadership team, and a set of local enumerators and interventionists met in Gaza to complete a 5-day training and reflexive discussion on the research design, assessment package, and program content. As a part of these meetings, the assessment package and intervention content were refined and in vivo roleplays and assessments were conducted to ensure mastery of the session content and study procedures. Following the 5-day intensive training, CRS leadership staff continued to meet with enumerators and interventionists to continue regular practice and address remaining questions, and the highest performing individuals were selected to continue as project staff.

### 4.3. Trial Design, Randomization, and Allocation Concealment

**Figure 1:**
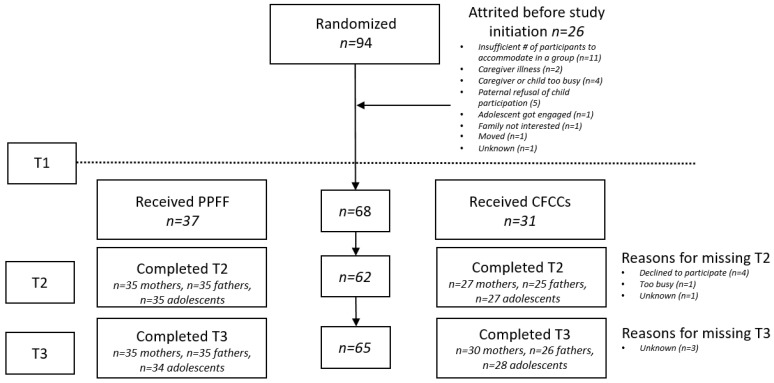
CONSORT Diagram.

This study was a pilot randomized controlled trial with a head-to-head parallel design for two interventions: *Child Friendly Community Centers* (CFCCs) and the *Promoting Positive Family Futures* program (PPFF). Families registered to receive psychosocial support services through CRS. All families were aware at the time of registration that they would be randomized to receive one of two types of support services. Following registration, families were assigned to either the PPFF program or the CFCCs. Assignments were conducted using a random number generator. *n* = 94 families were assigned to an intervention condition following registration. Of the randomized families, 26 attrited prior to the baseline interview (See Figure 1). As such, *n* = 68 proceeded to complete a baseline interview and receive services. Information regarding the reasons for family discontinuances can be found in Figure 1. The study was unblinded.

### 4.4. Implementation Timeline

Baseline interviews were conducted with all family members from October to November 2019. Of note, there was an escalation in conflict during the implementation of the baseline interview that project staff reported had significant implications for participant distress. As such, an indicator was recorded for those interviewed pre-escalation (0) and post-escalation (1), which was added a covariate to the models. Post-treatment assessments occurred from January to February 2020 and six-month follow-ups occurred from July to August 2020. Notably, the six-month follow-up was completed during the COVID-19 pandemic. As such, an abbreviated assessment package was delivered at this time and administered by phone. Both the baseline and post-test assessments were administered in person.

### 4.5. Interventions

***Promoting Positive Family Futures* (PPFF).** The PPFF program aims to help parents and their adolescent children develop emotional and cognitive awareness, learn constructive communication and conflict resolution strategies, and develop family-wide emotional security and positive family relationships. The PPFF program was co-developed in collaboration with the Palestinian Counseling Center (PCC) and Catholic Relief Services. First, the PIs and the PCC developed the preliminary framework for the PPFF program in a 3-day conference (2016), during which time the team met with several key Palestinian scholars, researchers in global mental health, and community leaders to discuss clinical needs and priorities in the region as they intersected with the proposed program framework. Following this, an open trial of a 6-session version of the program in the West Bank was conducted (2017–2018). After this, the PIs and the PCC team met in the West Bank to discuss the results and clinician and family feedback on the program. Together with colleagues from CRS, the teams then met to determine how best to integrate the feedback to fine-tune the program manual. The team also discussed how the manual and research design should be structured to be flexible to the diverse needs and experiences of families in the West Bank and Gaza. Changes to the PPFF manual as a part of this process included changes to the length (expanded to 8 sessions to include additional content on group trust and family relationship building) and structure (father sessions in separate, co-occurring groups; integration of an additional in-home session). Following this revision, the PIs traveled to Gaza to conduct a 5-day training on the program manual, incorporating final suggestions by local interviewers with expertise in psychosocial care (2019), including the expansion of sample discussion topics and prompts in the program manual. The PPFF program therefore reflects years of careful, long-term engagement, drawing from multiple sources including theoretical models in psychology, local empirical research, local clinical expertise, and iterative feedback from key informants.

The PPFF includes 8 sessions lasting approximately 1.5 hrs each, including a total of about 12 hrs of contact time Most sessions are group based (i.e., multiple families), but two sessions occur with each family individually, in-home. One in-home session focuses on the marital dyad, and the other in-home session includes the full triad (parents and adolescent child). Based on feedback from the pilot study in the West Bank, father groups meet separately from mother and adolescent groups; the content of both groups is identical and runs in parallel, such that all family members are receiving the same content at the same time. Thus, participants in the PPFF condition participated in 8 weeks of treatment, which included approximately 12 hrs of contact time for each family member. The fidelity of the interventionists to session content was regularly assessed by an external observer; a total of 20% of intervention sessions were assessed for fidelity. Fidelity was 99.6%.

*Session 1: Trust building.* The first session of the PPFF program is designed to introduce the basic program units to families, with the goal of building alliance and trust between group leaders and participants as well as among participants. Based on the recommendation of families and local partners, and consistent with social ecological theories of resilience that underscore the importance of cultural resources [40], this session centralizes its focus on a shared, enjoyable cultural activity. Facilitators are encouraged to frame the activity as an expression of *sumud*, or the maintenance of positive relationships and cultural practices as a way of demonstrating resistance to the occupation, which actively attempts to undermine such activities [25]. In addition to this shared activity, facilitators address questions and concerns about the program and establish the norms for group interaction (e.g., privacy, timeliness in arrival).

*Session 2: Introducing the Group.* In this session, the facilitators give a brief overview of the three theoretical bases of the program, and how they are interrelated. Specifically, multisystemic perspectives on resilience are discussed, emphasizing the embeddedness of individuals within family and community. Critical to this session is a recognition that distress in light of the ongoing violence of the occupation is a *normal* response to the events that are occurring and that psychological care in this context, while supporting families, should not be construed as palliative. Facilitators introduce individual experiences of managing stressful events from within a cognitive behavioral framework, identifying ways in which coping, communication, and distress are interrelated with the quality and security of family relationships.

*Session 3: In-home Parent Session.* This session was added following the open trial to achieve two key goals related to both treatment process and content. First, the session is designed to facilitate parent engagement and commitment to the overall treatment program by providing an overview of content from the *Introducing the Group* session in a private environment where parents can express any questions or concerns they might have about the program or approach. Second, the in-home parent session seeks to teach parents constructive conflict skills prior to their introduction in the broader group. Specifically, parents are taught a four-step method drawn from previous parent-adolescent communication programs [41] that includes: (1) identifying feelings using “I” statements; (2) pacing oneself and taking one point at time; (3) active listening to the other person’s perspective; and (4) indicating understanding of what the other person has shared.

*Session 4: Emotion Identification and Regulation.* Emotion identification skills represent an important pre-condition for engagement in cognitive behavioral approaches to care, communication about conflict (see Session 3), and are also associated with more positive affect and higher social support in adolescents [42]). As such, this session seeks to establish a transversal skill that is leveraged across sessions. First, families discuss the idea of complex/mixed emotions and the types of emotions that can be elicited by traumatic events, discussing how traumatic events and emotional experiences affect family relationships. Group members practice emotion identification skills and practice using of a subjective units of distress scale. Skills are practiced using vignettes, group activities, and individual worksheets. For homework, participants are given a mood log, the first line of which is completed in-session to ensure comprehension.

*Session 5: Improving Thoughts for Better Health in the Context of Conflict.* The fifth session begins with psychoeducational content on the effects of trauma on cognition, and the interrelations between thoughts, feelings, and behaviors. The group leaders work with participants to identify examples of situations relevant to their lives and then discuss several examples of how situations may elicit cascades of coordinated thoughts/emotions/behaviors. Families then discuss the relationship of these internal processes to interrelational processes, including family conflict. Examples from this conversation are used to bridge into a conversation about cognitive traps and restructuring, with a focus on querying the evidence to support potentially distorted thoughts. Of great importance to this session is the importance of recognizing the broader social context, as some thoughts that may represent distortions of actual risk in settings not affected by chronic overt violence may represent accurate assessments of risk in conflict-affected settings and serve important protective roles for health and safety. As such, group leaders are explicitly trained to be careful not to dismantle negative cognitions that are also *not* distortions.

*Session 6: Constructive Communication with Family Members.* The sixth session begins with a brief review of the content of the previous two sessions, linking individual coping with relational support and functioning. The session then provides psychoeducation about constructive and destructive forms of interpersonal conflict and aims to reinforce the four-step approach to communication introduced to couples in Session 3, with a greater focus on family-wide communication, including with adolescents. Role plays and conversation are used to facilitate skills practice and group leaders move around the room to give direct feedback to participants. Finally, the session closes by discussing the impact of the occupation on family relationships, including how family members can support one another.

*Session 7: Closing and Goodbye.* As the final group session, this session reviews key learnings of each of the core units (Sessions 2–6) and discusses the relation of key concepts from the program. Finally, and drawing upon the guiding framework of resilience theory, participants identify hopes for their future, hopes for their family, and work together to identify resources in their community that represent strengths and assets that they could access when needed. Group leaders close by reviewing these resources and facilitating a broader discussion of the relation between mental health, nonviolent resistance, and positive futures for families and communities.

*Session 8: Review Session Content and Progress.* This in-home session offers an opportunity for group leaders to meet individually with families to review all the skills learned in the program, discuss any concerns about their implementation, and if necessary, for group leaders to provide feedback on the use of skills in the home environment.

***Child Friendly Community Centers* (CFCCs).** In order to provide a robust test of effectiveness, the current study evaluates the PPFF as compared with a pre-existing psychosocial support program for adolescents in Gaza, *Child Friendly Community Centers* (CFCCs), a locally well-established program (treatment as usual; TAU). The CFCCs are comprised of a 25-session, adolescent-only support group. This program has limited parent engagement (1 optional conjoint session). The curriculum includes information on conflict resolution and problem solving, managing emotions, self-confidence, relationships with peers, human rights, and tolerance. Groups typically include between 12 and 15 adolescents. Sessions may be delivered in any format (e.g., weekly, bi-weekly, intensive workshops). In the current study, three sessions were delivered each week. Thus, in total, those adolescents assigned to the CFCCs participated in 8–9 weeks of treatment, which included a total of 50 hrs of contact time.

### 4.6. Measures

**Parental depression (T1, T2, T3).** Parental depression was assessed at each wave using the *Patient Health Questionnaire-9* (PHQ-9) [43]. The PHQ-9 has been regularly used in Arabic, including in Palestine, and has been established as a valid screening tool for internalizing disorders in the Middle East [44,45]. Respondents reports on the frequency of depressive symptoms using a scale of 0 (not at all) to 3 (nearly every day). Items are summed to create a total score, with higher scores representing higher levels of depression. Due to concerns about sensitivity, the final item of the PHQ-9, which asks about thoughts of death and self-harm was not administered. As such, the range of possible scores for this study was 0–24. Internal reliability was α = 0.78, 0.77, and 0.77 for fathers and α = 0.65, 0.68, and 0.71 for mothers.

**Parental emotion regulation (T1, T2).** Parental emotion regulation was assessed using the *Emotion Regulation Questionnaire* (ERQ) [46]. This 10-item assessment evaluates emotion regulation strategies in two domains: cognitive reappraisal; and expressive suppression. For each item, participants indicated the extent to which they agree or disagree, using a scale of 1 (strongly disagree) to 7 (strongly agree). Higher scores indicate higher levels of emotion regulation. The ERQ has been used in Arabic, with evidence of its validity and reliability in several Arabic-speaking countries [47]. In the current study, the internal reliability for the expressive suppression scale did not reach adequate reliability and was therefore not included in the analyses. Internal reliability for the emotion regulation using cognitive reappraisal subscale was marginal at baseline but was acceptable at post-test for both mothers and fathers. Internal reliability was α = 0.64 and 0.79 for fathers and α = 0.54 and 0.75 for mothers.

**Parent and adolescent emotional security (T1, T2, T3).** Parent and adolescent emotional security in the family was assessed using the *Security in the Family Scale* (SIFS) [48]). This scale assesses the extent to which individuals feel that their family environment is secure, dependable, and engaged. The scale has not been previously used in Arabic and as such, was forward- and back-translated by bilingual members of the research team. Discrepancies in the assessment or questions about semantic equivalence of wording were collaboratively discussed and resolved by the research team. For each of the 24 items on the scale, participants reported the extent of their agreement using a scale of 1 (completely disagree) to 4 (completely agree). Sixteen items are reversed scored, and higher total scores indicate higher security in the family systems. Internal reliabilities were: α = 0.75, 0.78, and 0.78 for fathers; α = 0.84, 0.77, and 0.75 for mothers; and α = 0.81, 0.74, and 0.75 for adolescents.

**Adolescent adjustment (T1, T2, T3).** Adolescent adjustment was assessed using the *Strengths and Difficulties Questionnaire* (SDQ) [49]. This 25-item scale evaluates youth emotional and behavioral adjustment in five domains: emotional symptoms (5 items); conduct problems (5 items); hyperactivity/inattention (5 items); peer relationship problems (5 items); and prosocial behavior (5 items). The first four domains are summed together to provide a total adjustment problems score, with higher values indicated more adjustment difficulties. In the current study, both mothers and fathers reported on child adjustment problems. The scale has been previously used and evaluated in Arabic, though caution in the extension of the subscales to non-Western groups has been noted, suggesting that a total adjustment problems score is likely most appropriate in the absence of further exploratory factor analyses [50,51]. As such, the current study used only the total adjustment score. Reliabilities were 0.67, 0.76, and 0.75 for paternal report and 0.67, 0.73, and 0.73 for maternal report.

**Adolescent resilience (T1, T2).** Adolescents provided self-report on resilience using the *Child and Youth Resilience Questionnaire* (Ungar & Liebenberg, 2011). This 25-item assessment evaluates multiple dimensions of resilience from a social ecological perspective (i.e., including personal, relational, and cultural/community strengths). It has been widely used around the world, and Palestinian samples were included in its initial development [52]. Internal reliabilities were α = 0.87 and 0.91.

### 4.7. Statistical Methods

All analyses were conducted in Stata 15.2 [53]. The first study aim, which examined within-group change from pre-test to post-test and from pre-test to six-month follow-up was evaluated using paired t-tests. Effects sizes were calculated using Cohen’s *d*, with descriptive cut-offs for 0.2, 0.5, and 0.8 for small, medium, and large effect sizes, respectively [54]. The second study aim, which examined between-group differences (PPFF vs. TAU) used path analysis to examine post-test and six-month follow-up outcomes by group, controlling for baseline levels of each outcome and timing of the baseline interview. Given that this study was a pilot trial, the current study was adequately powered only to detect large effect sizes between groups. Missing data were accounted for using full information maximum likelihood estimation. Finally, the third and fourth aims of the study, which sought to examine indirect treatment effects, used path modeling, with full information maximum likelihood to account for missing data. Joint significance tests were used to identify mediating pathways, and indirect effects were calculated to determine the magnitude of the mediating effects. For two path mediation effects (i.e., a→b→c), Monte Carlo methods were used to calculate the confidence interval of the effect, with a confidence interval not including zero indicating a significant indirect effect. For three path mediation effects (i.e., a→b→c→d), joint significance testing was used as the primary determinant of identifying potential mediation given that in this case, joint significance testing is more robust to Type I error than are Monte Carlo methods for estimating the magnitude of the indirect effect [55].

## 5. Results

Descriptive statistics for all reporters across time and group can be found in Table 1. There were no significant differences between groups on any study variable at baseline.

## 6. Within-Group Change

### 6.1. Parent Outcomes

**Parental Depression.** For fathers in the treatment group, depression significantly decreased between baseline and post-test (*t*(1, 35) = −6.23, *p* < 0.001, *d* = −1.24) and between baseline and follow-up (*t*(1, 32) = −2.93, *p* = 0.006, *d* = −0.43). This was also true of fathers in the TAU group, with significant declines in depression from baseline to post-test (*t*(1, 25) = −3.17, *p* = 0.004, *d* = −0.76) as well as from baseline to follow-up (*t*(1, 26) = −3.97, *p* < 0.001, *d* = −0.89).

For mothers in the treatment group, depression significantly decreased between baseline and post-test (*t*(1, 35) = −6.23, *p* < 0.001, *d* = −0.87) and between baseline and follow-up (*t*(1, 27) = −4.42, *p* < 0.001, *d* = −0.86). This was also true of mothers in the TAU group, with significant declines in depression from baseline to post-test (*t*(1, 27) = −4.24, *p* < 0.001, *d* = −0.71) as well as from baseline to follow-up (*t*(1, 26) = −3.46, *p* = 0.002, *d* = −0.77).

**Parental Emotion Regulation using Cognitive Reappraisal.** Emotion regulation using cognitive reappraisal increased from baseline to post-test for fathers in the treatment group (*t*(1, 34) = 4.60, *p* < 0.001, *d* = 1.05), but did not significantly increase for fathers in the TAU group. Similarly, mothers in the treatment group reported a significant increase in emotion regulation using cognitive reappraisal from baseline to post-test (*t*(1, 34) = 4.70, *p* < 0.001, *d* = 0.93), but the increase for mothers in the TAU was not statistically significant.

**Parental Emotional Security in the Family.** Father emotional security in the family significantly increased from baseline to post-test for fathers in the treatment group (*t*(1, 34) = 3.81, *p* < 0.001, *d* = 0.50), but not for fathers in the TAU group. Neither fathers in the treatment nor fathers in the TAU group demonstrated increases in emotional security in the family from baseline to six-month follow-up.

In contrast, mothers in both the treatment and TAU conditions reported higher emotional security in the family at post-test as compared to baseline (*t*(1, 34) = 5.68, *p* < 0.001, *d* = 0.96; *t*(1, 24) = 6.10, *p* < 0.001, *d* = 0.87, respectively). Mothers in both groups also reported higher scores from baseline to six-month follow-up (*t*(1, 34) = 2.68, *p* = 0.011, *d* = 0.57; *t*(1, 24) = 2.14, *p* = 0.041, *d* = 0.35, for the treatment and TAU respectively).

### 6.2. Adolescent Outcomes

**Adolescent Adjustment.** Father-reported adolescent adjustment problems decreased from baseline to posttest for both the treatment (*t*(1, 35) = 7.39, *p* < 0.001, *d* = −1.23) and the TAU group (*t*(1, 25) = 6.78, *p* < 0.001, *d* = −1.12). The same was true for maternal report of adolescent adjustment problems, with significant within-group improvements for both the treatment (*t*(1, 34) = 3.43, *p* = 0.001, *d =* −0.74) and the TAU groups (*t*(1, 27) = 4.81, *p* < 0.001, *d =* −0.81) from baseline to post-test. The same pattern of effects was noted from baseline to six-month follow-up, with mother and fathers in both groups reporting significant improvement in symptoms (Paternal report, PPFF [*t*(1, 34) = −3.86, *p* < 0.001, *d* = −0.66]; Paternal report, TAU [*t*(1, 25) = −3.01, *p* = 0.005, *d* = 0.62]; Maternal report, PPFF [*t*(1,34) = −3.48, *p* = 0.001, *d* = −0.64]; Maternal report, TAU [*t*(1, 28) = 4.35, *p* < 0.001, *d* = −0.87).

**Adolescent Resilience.** Adolescents in the treatment group reported significant increases in their resilience from baseline to post-test (*t*(1, 34) = 2.26, *p* = 0.030, *d* = 0.39) as did adolescents in the TAU group (*t*(1, 26) = 3.72, *p* < 0.001, *d* = 0.81). Adolescent resilience was not collected at the six-month follow-up.

**Adolescent Security in the Family.** Adolescents in the treatment group reported significant increases in their security in the family from baseline to post-test (*t*(1, 34) = 3.21, *p* = 0.002, *d* = 0.55) as did adolescents in the TAU group (*t*(1, 34) = 2.65, *p* = 0.014, *d* = 1.06). There were no significant differences in adolescent security in the family at six-month follow-up, for either the treatment or the TAU group.

## 7. Between-Group Change

### 7.1. Parent Outcomes

**Parental Depression.** Controlling for baseline depression, emotion regulation and timing of the interview, participation in the PPFF was associated with significantly lower depression for fathers at the post-test (*β* = −0.26, *p* = 0.043). There were no significant differences between groups at six-month follow-up. There were also no significant differences between the PPFF and TAU groups in maternal depression.

**Parental Emotion Regulation using Cognitive Reappraisal.** Controlling for baseline depression, emotion regulation using cognitive reappraisal and timing of the interview, participation in the PPFF was associated with significantly higher levels of emotion regulation using cognitive reappraisal for fathers at the post-test (*β* = 0.38, *p* = 0.002). Controlling for baseline emotion regulation using cognitive reappraisal and timing of the interview, participation in the PPFF was also associated with significantly higher levels of emotion regulation using cognitive reappraisal for mothers at the post-test (*β* = 0.33, *p* = 0.005).

**Parental Emotional Security in the Family.** There were no significant differences in parent-reported emotional security in the family between groups for either mothers or fathers at post-test or six-month follow-up.

### 7.2. Adolescent Outcomes

**Adolescent Adjustment.** There were no significant differences in either mother-reported or father-reported adolescent adjustment between groups at post-test or six-month follow-up.

**Adolescent Resilience.** There were no significant differences between groups in adolescent resilience at post-test.

**Adolescent Emotional Security in the Family.** There were no significant differences between groups in adolescent emotional security in the family at post-test or six-month follow-up.

## 8. Indirect Effects of Intervention

### 8.1. Indirect Effects of Treatment on Parental Depression via Emotion Regulation

In order to evaluate the effects of treatment through a key hypothesized mechanism of change, the indirect effects of treatment on depression via emotion regulation using cognitive reappraisal were examined. That is, we examined the effects of treatment on emotion regulation using cognitive reappraisal at post-test. In turn, we examined the effects of emotion regulation using cognitive reappraisal on depression at post-test (i.e., PPFF→emotion regulation→depression). For fathers, participation in the PPFF program was association with significantly higher levels of emotion regulation using cognitive reappraisal at post-test (*β* = 0.36, *p* = 0.004), which was in turn associated with lower levels of depression at the same time point (*β* = −0.71, *p* < 0.001). This indirect effect was significant (IE: −0.26, 95% CI: −0.45, −0.09). Additionally, paternal depression at post-test was significantly associated with paternal depression at six-month follow-up (*β* = 0.44, *p* = 0.002). See Table 2 for additional detail.

This pattern of effects was partially replicated for mothers, with treatment associated with improved emotion regulation using cognitive reappraisal at post-test (*β* = 0.33, *p* = 0.003), which was in turn associated with depression at the same time point (*β* = −0.31, *p* = 0.001). This indirect effect was significant (IE: −0.10, 95% CI: −0.205, −0.026). Maternal depression at post-test, however, was not significantly associated with depression at six-month follow-up (*β* = 0.27, *p* = 0.065). See Table 3 for additional detail.

### 8.2. Indirect Effects of Treatment on Adolescent Adjustment via Parental Depression

No paths examining the association between paternal depression and emotion regulation using cognitive reappraisal at post-test with father-reported adolescent adjustment at six-month follow-up were significant. In contrast, maternal emotion regulation at post-test was significantly associated with mother-reported adolescent adjustment at the six-month follow-up (*β* = −0.26, *p* = 0.047). The indirect effect of treatment on adolescent adjustment via improvements in maternal emotion regulation using cognitive reappraisal were significant (IE: −1.15, 95% CI: −2.48, −0.16). The total effect (i.e., the direct and indirect effect together) of PPFF, however, was not significantly different from zero given the opposite direction of the coefficients, indicating inconsistent mediation (See Table 4). Such results indicate the presence of possible suppressor or confounding effects that could be better explored in future research with larger samples.

## 9. Discussion

The current study aimed to address a significant gap in the research literature by evaluating a brief, family-based intervention—the *Promoting Positive Family Futures* (PPFF) program—conducted in Gaza City, Gaza. The program was collaboratively designed by multiple constituents with the aim of supporting both the individual mental health of family members and family-wide emotional security and communication. Using a randomized control design, *n =* 68 families were randomized between two different treatment conditions and evaluated across three time points of assessment (pre-test [T1], post-test [T2] and six-month follow-up [T3]. The sample demonstrated very low attrition after participants matriculated in the study (96% retention from pretest to the six-month follow-up) even in the context of conflict escalation and the COVID-19 pandemic. The current study aimed to evaluate: (1) within-group change over time; (2) between-group differences in treatment effects; and (3) indirect effects of treatment via parental emotion regulation and mental health.

Regarding parental emotional security in the family, mothers in both groups reported significant improvements over time, with effect sizes ranging from small to large, depending upon the time point and condition. For fathers, within-group analyses found that emotional security in the family only increased for those participating in the PPFF group; the examination of between group differences was not significant, but this could be due to the fact that the sample size of the current study was small, and the effect size of the within-group effect was medium (*d* = 0.50). This is very promising given that paternal engagement in MHPSS—and in psychological research more broadly—has been understudied [10]. The current study suggests that not only is it feasible for programs to include fathers, but also that their inclusion has great potential benefit for both them and the family system at large. Importantly, however, these positive effects were not sustained for fathers in either condition at the six-month follow-up, either in the within- or between-group analyses. Notably, the six-month follow-up was conducted in the context of the COVID-19 pandemic, which may have resulted in a mitigation of the positive effects of treatment at this time point.

Mothers and fathers in both groups experienced significant declines in depressive symptoms—effects that were large in magnitude. Between-group analyses indicated that the difference between groups was not significant for mothers but that fathers in the PPFF condition had significantly better improvement in their symptoms of depression than did fathers in the TAU group. Both within- and between-group analyses also indicated that parents in the PPFF group had significantly better levels of emotion regulation using cognitive reappraisal at post-test than did parents in the TAU group, who did not experience significant positive change on this dimension. Moreover, path models testing indirect effects demonstrated that parent emotion regulation using cognitive reappraisal mediated the relationship between PPFF participation and depression at post-test, suggesting that the PPFF contributes to important mechanisms of change for parental mental health.

For adolescents, no differences emerged between groups on any dimension of assessment. Rather, adolescents in both groups evidenced significant improvements in adjustment problems (medium to large effect sizes), resilience (small to large effect sizes), and security in the family (medium to large effect sizes). Importantly, however, the PPFF program was uniquely associated with indirect benefits for adolescent adjustment at the six-month follow-up via maternal emotion regulation using cognitive reappraisal.

Interestingly, support for mechanisms of improvement based on the PPPF intervention was most evident for emotional regulation using cognitive reappraisal, and improvements in continua of depressive symptomatology. Notably, these processes are emphasized as fundamental to family-wide adjustment and well-being in all three theoretical models underlying the PPFF program. While not all potential mechanisms of change were evaluated in this pilot study, the findings of this evaluation of the PPFF intervention indicate the strength of the integration of the three theoretical frameworks in the PPFF intervention to synergistically influence common transdiagnostic processes associated with improvements in the well-being of multiple family members (i.e., mothers, fathers, and adolescents).

## 10. Limitations and Future Directions

Although the study had several strengths, some limitations should also be noted. First, the current study was not able to evaluate the effects of treatment dosage relative to each program. That is, the current study used only one post-test assessment following the completion of the intervention in both conditions. Moreover, given that the great majority of families starting the program completed all sessions, there was little within-condition variability in attendance. As such, we could not evaluate dosage effects within condition, though the high attendance rate is a promising marker of feasibility and acceptability. The comparison of PPFF to a well-established and locally successful TAU is a strength of this research design. The results provided cogent evidence for program efficacy, both in terms of enhanced benefits and greater sustainability. Further, this approach is optimal from a research design perspective, as compared with a common practice of comparisons with a no treatment control group, which are only appropriate in the context of no clearly effective alternative paradigm. In order to better delineate the specific effects of each program, and provide more information on the effect of dosage, however, future research should consider dual post-tests, one that permits a comparative assessment of the programs after equivalent dosage (i.e., after eight sessions), and one that follows completion of the program to test the full effectiveness of the program package.

In addition, the current study did not include any observational assessments of parent-adolescent or interparental interactions. Such data will likely be valuable information in evaluating hypothesized mechanisms of change for family-wide emotional security and will provide a more direct assessment of skills targeted in the program. It may be that the positive benefits of the PPFF on family emotional security occur indirectly via changes in family-wide communication and that more robust assessments of family functioning over longer periods of time will give better insight into intrafamilial processes following treatment.

Finally, although the current study employed a rigorous design, the sample size was limited for some statistical purposes, but is nonetheless substantial given both the context and the fact that the study involved long-term follow-up. Because of this, the number of pathways and covariates that could be included in models were limited, and the ability to detect small effect sizes was insufficient. Given that these preliminary findings demonstrate promising effectiveness, future research should consider bringing the PPFF program to scale, to gain greater insight and precision into estimates and domains of effectiveness.

## 11. Clinical and Public Health Implications

The current study has several important clinical and public health implications. Specifically, findings suggest the importance of the engagement of multiple family members in MHPSS services. Practically, our learnings throughout collaborative design and implementation also suggest the importance of gathering community, clinician, and stakeholder feedback in promoting family engagement in psychosocial support programs that are consistent with cultural and social norms around mental health and family life. The initial trial of this program in the West Bank, for example, included fathers in the broader group with mothers and adolescents, and fathers’ participation in this format was almost nonexistent. After receiving family feedback, the PPFF program was modified to include a separate co-occurring group for fathers, which resulted in 100% participation of fathers in the current study.

Practically, the PPFF approach triples the number of potential beneficiaries and reduces the implementation timeline for delivery to a third of the number of sessions to establish effectiveness, and less than a fourth of the number of hours of contact time. Substantively, the broader engagement of family members attends to the reality that all family members living in conflict settings are affected by the violence they experience, and that care for children and adolescents is likely to be positively impacted by concurrent supports for parents [19,56]. This hypothesis was supported by the data, with adolescent adjustment outcomes at six months associated with the influence of PPFF on parent emotion regulation using cognitive reappraisal at post-test.

Further, the PPFF program was able to establish promising evidence of effectiveness and identify potential mechanisms of change. In the West Bank and Gaza, rigorous evaluations of psychological interventions have had difficulty establishing effectiveness ([18]. Unfortunately, the limited effectiveness for MHPSS programs for children and youth is not limited to the West Bank and Gaza. Rather, MHPSS programs across multiple global contexts have been found to have modest effects [8], and few RCTs in conflict-affected settings include an evaluation of both treatment efficacy and mechanisms of change [7].

It is also important to note that the theoretical models undergirding the PPFF program have garnered widespread empirical support across multiple global contexts. As such, the program is agile for cross-contextual relevance and adaptation. Given the large number of children and families living in conflict-affected settings, the need is great, and the PPFF provides one potential resource for clinicians and organizations delivering MHPSS.

## 12. Conclusions

Overall, study findings indicated that the PPFF—a theoretically grounded, brief, family-based intervention—was equally effective as compared to a longer, well-established program in many domains, and was more effective in some domains, particularly those related to parental functioning. These findings suggest a key advance in the MHPSS field, both in the rigorous evaluation of a family-based intervention [9] and the demonstrable effectiveness of this program relative to an established MHPSS support focused on adolescent-only care. In addition, the results of the current study demonstrate preliminary evidence for the hypothesized mechanisms of treatment change. That is, emotion regulation using cognitive reappraisal is a key transdiagnostic mechanism underlying psychological distress [32,57]. Thus, not only does this study contribute to the overall body of work regarding the effectiveness of MHPSS for adolescents and families, but it also makes a significant contribution to the research literature in identifying key mechanisms of treatment change for parents and adolescents [7]. In conclusion, this study reflects an innovative approach to advancing evidence-based supports for adolescents and families living in conflict-affected settings, thereby addressing an important gap in the global literature, with clear broader relevance for conflict-affected settings worldwide, including settings affected by chronic violence in the United States.

## Figures and Tables

**Table 1:** Descriptive statistics for study measures, by group and across time.

	Baseline		Post-Test		3-Months	
	PPFF M (SD)	CFCC M (SD)	PPFF M (SD)	CFCC M (SD)	PPFF M (SD)	CFCC M (SD)
*Fathers*						
Depression	11.19 (5.17)	12.00 (5.37)	5.72 (3.31)	8.00 (5.11)	9.05 (4.91)	8.38 (4.58)
Emotion Regulation	30.61 (6.10)	30.23 (5.58)	35.78 (4.13)	32.17 (5.81)	--	--
Emotional Security	87.98 (9.79)	88.50 (9.82)	93.73 (10.13)	91.01 (10.44)	88.97 (8.67)	89.65 (9.87)
*Mothers*						
Depression	12.27 (4.27)	11.16 (5.02)	8.77 (3.99)	8.03 (4.21)	8.71 (4.32)	7.83 (3.86)
Emotion Regulation	28.46 (5.74)	28.71 (6.82)	33.60 (5.20)	30.73 (5.89)	--	--
Emotional Security	77.98 (11.85)	81.69 (9.87)	88.64 (10.28)	91.89 (10.94)	83.89 (8.76)	86.70 (10.94)
*Adolescents*						
Adjustment—Father report	17.11 (4.79)	15.35 (5.74)	10.46 (5.60)	9.64 (4.56)	13.77 (4.69)	11.96 (4.55)
Adjustment—Mother report	18.41 (5.99)	17.90 (6.09)	14.06 (5.98)	13.48 (5.58)	14.86 (5.47)	12.67 (5.94)
Emotional Security	86.86 (11.76)	87.35 (13.71)	92.50 (10.16)	91.59 (9.48)	87.98 (9.37)	85.82 (8.09)
Resilience	3.97 (0.48)	3.80 (0.60)	4.16 (0.53)	4.18 (0.50)	--	--

**Table 2:** Indirect effects model: Fathers’ emotion regulation and depression.

	*β* (SE)	*p*	95% CI
*Emotion Regulation T2*			
PPFF	0.36 (0.12)	0.004	0.12, 0.61
Depression T1	−0.20 (0.14)	0.148	−0.47, 0.07
Emotion Regulation T1	0.14 (0.13)	0.305	−0.12, 0.40
Pre or Post Escalation	0.09 (0.10)	0.355	−0.10, 0.28
*Depression T2*			
PPFF	0.03 (0.10)	0.765	−0.17, 0.23
Depression T1	0.21 (0.08)	0.013	0.04, 0.37
Emotion Regulation T2	−0.71 (0.09)	<0.001	−0.89, −0.54
Pre or Post Escalation	−0.06 (0.09)	0.481	−0.24, 0.11
*Depression T3*			
PPFF	0.18 (0.11)	0.099	−0.03, 0.40
Depression T2	0.44 (0.14)	0.002	0.16, 0.72
Emotion Regulation T2	−0.12 (0.16)	0.452	−0.44, 0.20

**Table 3:** Indirect effects model: Mothers’ emotion regulation and depression.

	*β* (SE)	*p*	95% CI
*Emotion Regulation T2*			
PPFF	0.33 (0.11)	0.003	0.12, 0.55
Depression T1	−0.20 (0.11)	0.079	−0.42, 0.02
Emotion Regulation T1	0.33 (0.10)	0.001	0.14, 0.51
Pre or Post Escalation	0.13 (0.13)	0.349	−0.14, 0.39
*Depression T2*			
PPFF	0.11 (0.13)	0.389	−0.14, 0.36
Depression T1	0.52 (0.08)	<0.001	0.35, 0.68
Emotion Regulation T2	−0.31 (0.09)	0.001	−0.49, −0.13
Pre or Post Escalation	−0.01 (0.08)	0.952	−0.35, 0.68
*Depression T3*			
PPFF	0.12 (0.13)	0.332	−0.02, 0.55
Depression T2	0.27 (0.14)	0.065	−0.02, 0.14
Emotion Regulation T2	−0.13 (0.14)	0.338	−0.13, 0.38

**Table 4:** Indirect effects model: Adolescent adjustment via maternal outcomes.

	*β* (SE)	*p*	95% CI
*Emotion Regulation T2*			
PPFF	0.32 (0.11)	0.004	0.10, 0.54
Depression T1	−0.20 (0.11)	0.075	−0.42, 0.02
Emotion Regulation T1	0.32 (0.09)	0.001	0.13, 0.51
Pre or Post Escalation	0.13 (0.13)	0.345	−0.14, 0.39
*Depression T2*			
PPFF	0.11 (0.13)	0.404	−0.14, 0.36
Depression T1	0.52 (0.08)	<0.001	0.35, 0.68
Emotion Regulation T1	0.03 (0.11)	0.760	−0.18, 0.25
Pre or Post Escalation	0.002 (0.09)	0.981	−0.18, 0.68
Emotion Regulation T2	−0.31 (0.09)	0.001	−0.49, −0.13
*Adolescent Adjustment T3*			
PPFF	0.27 (0.09)	0.007	0.08, 0.47
Depression T2	−0.11 (0.11)	0.341	−0.32, 0.11
Emotion Regulation T2	−0.31 (0.13)	0.015	−0.57, −0.06
Adolescent Adjustment T1	0.40 (0.11)	<0.001	0.19, 0.61

## Data Availability

The data are not publicly available as participants did not consent to this. Data are available on request and with the approval of participating agencies, with appropriate measures taken to protect the privacy of participants. Please contact the corresponding author with any requests for information.
